# Prevalence of chronic pain in Libya before and after the uprising of 17 February 2011

**DOI:** 10.3402/ljm.v8i0.20125

**Published:** 2013-02-08

**Authors:** Osama A. Tashani

**Affiliations:** Faculty of Health and Social Sciences, Leeds Metropolitan University, UK

Two questionnaires were translated into Arabic and culturally adapted to measure chronic pain and neuropathic pain in the general population in Derna, Libya (1, 2), and then in a country-wide survey. A point prevalence of chronic pain and neuropathic pain in Libya was determined. The findings of this series of studies suggested that the prevalence of chronic pain in Libya, which was 19.6%, is similar to the average European estimate (3) despite the cultural dissimilarities of the two regions (4) and the environmental differences surrounding the surveyed samples.

The studies of chronic pain in Libya conducted by our pain research team, which involved a PhD student sponsored by the Libyan High Education Authority, indicated that Libyan women were found to have more chronic pain conditions than men. These sex and gender differences in chronic pain highlighted a general trend in epidemiological studies in many parts of the world (5).

However, this programme of studies on prevalence of chronic pain was conducted just before the uprising of 17 February 2011 in Libya, and one should be cautious about the validity of some of the findings’ implications for immediate pain management priorities in the country. For example, the findings of the pre-conflict survey that being an old woman with children carries double the risk of having a chronic pain condition compared to a young person with no children may have changed as a result of the war which claimed the lives of around 30,000 Libyans and caused serious injuries to more than 20,000 young men.

There is an urgent need for a new programme of studies using the PRIME approach on prevalence, impact, and economic cost of chronic pain (6) in post-conflict Libya. This should inform the health authorities in Libya and help plan management of chronic pain conditions resulting from the conflict. This will also serve as a model that can be applied to other countries from the developing world experiencing similar conflicts (7).

*Osama A. Tashani* Faculty of Health and Social Sciences Leeds Metropolitan University, UK Email: osamatashani@yahoo.com

## References

[1] Elzahaf RA, Tashani OA, Unsworth BA, Johnson MI (2012). Translation and linguistic validation of the Self-completed Leeds Assessment of Neuropathic Symptoms and Signs (S-LANSS) scale for use in a Libyan population. Pain Pract.

[2] Elzahaf RA, Tashani OA, Johnson MI (2012). Prevalence of chronic pain among Libyan adults in Derna city: a pilot study to assess the reliability, linguistic validity and feasibility of using an Arabic version of the structured telephone interviews questionnaire on chronic pain. Pain Pract.

[3] Breivik H, Collett B, Ventafridda V, Cohen R, Gallacher D (2006). Survey of chronic pain in Europe: prevalence, impact on daily life, and treatment. Eur J Pain.

[4] Alabas OA, Tashani OA, Johnson MI (2012). Effects of ethnicity and gender role expectations of pain on experimentally induced pain: a cross-cultural study between Libyan and white British students. Eur J Pain.

[5] Tashani OA, Alabas OAM, Johnson MI (2012). Understanding the gender-pain gap. Editorial. Pain Manag.

[6] Raftery MN, Sarma K, Murphy AW, De la Harpe D, Normand C, McGuire BE (2011). Chronic pain in the Republic of Ireland—community prevalence, psychosocial profile and predictors of pain-related disability: results from the Prevalence, Impact and Cost of Chronic Pain (PRIME) study, part 1. Pain.

[7] Charlson FJ, Steel Z, Degenhardt L, Chey T, Silove D, Marnane C, Whiteford HA (2012). Predicting the impact of the 2011 conflict in Libya on population mental health: PTSD and depression prevalence and mental health service requirements. PLoS One.

